# Supplementary material for: Evaluation of Genomic Typing Methods in the Salmonella Reference Laboratory in Public Health, England, 2012–2020
Source: Pathogens. 2023 Jan 31;12(2):223. doi: 10.3390/pathogens12020223 (PMC9966477; doi:10.3390/pathogens12020223)
Supplement: Supplementary file 1 [file pathogens-12-00223-s001.zip › Supplementary information - Technical information - Merging of Clusters.pdf]

Supplementary Information

Merging of Clusters

Although WGS has made a notable contribution the way we detect clusters, it is important to understand limitations and parameter considerations in any methodology. Once such challenge with the SNP addresses is as datasets and representation of the strain population increases, you can get the merging of clusters. When new isolates are added to the Snapper database of each eBG [1], if they are within a SNP threshold (i.e. 5-SNPs) from two separate clusters which were previously unlinked at the 5-SNP level, these two clusters will merge to include this new isolate. This results in all of the isolates of the old and new clusters as well as the new isolate which has 'linked' them adopting a single SNP address (the numerically lowest one); this is called a SNP cluster merge. These merges and subsequent SNP address changes are a function of the use of single linkage clustering and while this changes the membership of the isolates with a given SNP address. The change of SNP address does not change the genetic relatedness of the isolates; their phylogenetic relationship remains the same and it is for this reason the phylogeny is utilised for assessing genetic relationships between isolates during outbreak investigations. Two examples of merged clusters include the *S*. Typhimurium, 1.2.3.175.175.175.x outbreak associated with eggs from a Polish producer [2,3] and the *S*. Enteritidis 1.2.3.38.38.38.x outbreak associated with eggs from a German producer [4-8]. A merge in relation to these clusters have now occurred and the SNP addresses have subsequently changed (1.2.3.18.175.175.x and 1.2.3.18.38.38.x respectively) (Table S1). Merging of clusters are managed with prospective reporting of merged clusters with every batch of data and updating our SNP cluster analysis in relation to any change.

Principles of the maximum SNP distance within Single Linkage Clusters (SLC)

As well as merging of clusters, consideration is needed when using SLC reside in long persistent clusters and accumulation of SNPs. SLC is based on grouping isolates in agglomerative clustering, each new isolate will be aggregated to its closest pair. If the closest isolate is part of an existing cluster, the cluster is then expanded with the new isolate added. It results in clusters where isolates at opposite ends of the clusters having larger distance than the initial cluster level threshold.

If an initial cluster was detected at 5 SNPs and isolates were originally all within 5 SNPs of each other, then addition of new isolates overtime can lead to an increase of the maximum distance

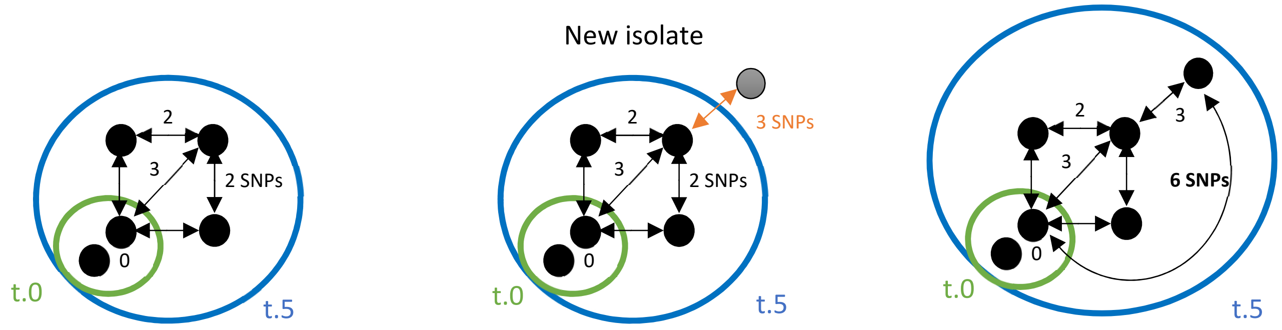

Initial cluster t5
Min 0 SNPs
Max 3 SNPs

Curent cluster t5
Min 0 SNPs
Max 6 SNPs

within a cluster (see above figures). New SNPs within related isolates could be explained by mutation of strains across time or selective pressure within the environment.

The assessment of clusters is always made by looking into the SNP address derived by SLC followed up by phylogenetic reconstruction as the phylogeny will show the topology/placement of isolates within a clusters. This is to ensure, cluster with exceeding maximum distance can still be interpreted using SNP addresses or if they need more bioinformatics context for interpreatation.

One example of a cluster that was initially defined at t5 but as has become larger over time is the example of the *S*. Enteritidis 1.2.3.18.175.175.% cluster (Table S1), which contains over 800 isolates with a maximum distance of 20 SNPs. These types of clusters pose a challenge to interpret, but integrated approaches of epidemiological evidence, microbiology, genomics and phylogeny are useful to interpret context.

Recombination

Most of organisms in bacterial kingdom have the possibilities of rearranging their genomes, include extra DNA in terms of mobile genetics elements (phages, prophages, plasmids integration…). Within UKHSA, each new outbreak has regions of mobile elements removed.

References for Supplementary information

1. Dallman, T.; Ashton, P.; Schafer, U.; Jironkin, A.; Painset, A.; Shaaban, S.; Hartman, H.; Myers, R.; Underwood, A.; Jenkins, C.; et al. SnapperDB: a database solution for routine sequencing analysis of bacterial isolates. *Bioinformatics* **2018**, *34*, 3028-3029, doi:10.1093/bioinformatics/bty212.
2. Coipan, C.E.; Dallman, T.J.; Brown, D.; Hartman, H.; van der Voort, M.; van den Berg, R.R.; Palm, D.; Kotila, S.; van Wijk, T.; Franz, E. Concordance of SNP- and allele-based typing workflows in the context of a large-scale international Salmonella Enteritidis outbreak investigation. *Microbial genomics* **2020**, *6*, doi:10.1099/mgen.0.000318.
3. Pijnacker, R.; Dallman, T.J.; Tijsma, A.S.L.; Hawkins, G.; Larkin, L.; Kotila, S.M.; Amore, G.; Amato, E.; Suzuki, P.M.; Denayer, S.; et al. An international outbreak of Salmonella enterica serotype Enteritidis linked to eggs from Poland: a microbiological and epidemiological study. *Lancet Infect Dis* **2019**, *19*, 778-786, doi:10.1016/s1473-3099(19)30047-7.
4. Dallman, T.; Inns, T.; Jombart, T.; Ashton, P.; Loman, N.; Chatt, C.; Messelhaeusser, U.; Rabsch, W.; Simon, S.; Nikisins, S.; et al. Phylogenetic structure of European Salmonella Enteritidis outbreak correlates with national and international egg distribution network. *Microb Genom* **2016**, *2*, e000070, doi:10.1099/mgen.0.000070.
5. Hörmansdorfer, S.; Messelhäußer, U.; Rampp, A.; Schönberger, K.; Dallman, T.; Allerberger, F.; Kornschober, C.; Sing, A.; Wallner, P.; Zapf, A. Re-evaluation of a 2014 multi-country European outbreak of Salmonella Enteritidis phage type 14b using recent epidemiological and molecular data. *Eurosurveillance* **2017**, *22*, 17-00196, doi:doi:https://doi.org/10.2807/1560-7917.ES.2017.22.50.17-00196.
6. Inns, T.; Lane, C.; Peters, T.; Dallman, T.; Chatt, C.; McFarland, N.; Crook, P.; Bishop, T.; Edge, J.; Hawker, J.; et al. A multi-country Salmonella Enteritidis phage type 14b outbreak associated with eggs from a German producer: 'near real-time' application of whole genome sequencing and food chain investigations, United Kingdom, May to September 2014. *Eurosurveillance* **2015**, *20*, 21098, doi:doi:https://doi.org/10.2807/1560-7917.ES2015.20.16.21098.
7. Quick, J.; Ashton, P.; Calus, S.; Chatt, C.; Gossain, S.; Hawker, J.; Nair, S.; Neal, K.; Nye, K.; Peters, T.; et al. Rapid draft sequencing and real-time nanopore sequencing in a hospital outbreak of Salmonella. *Genome Biol* **2015**, *16*, 114, doi:10.1186/s13059-015-0677-2.
8. Pearce, M.E.; Alikhan, N.F.; Dallman, T.J.; Zhou, Z.; Grant, K.; Maiden, M.C.J. Comparative analysis of core genome MLST and SNP typing within a European Salmonella serovar Enteritidis outbreak. *Int J Food Microbiol* **2018**, *274*, 1-11, doi:10.1016/j.ijfoodmicro.2018.02.023.
