# Supplementary material for: Evaluation of Genomic Typing Methods in the Salmonella Reference Laboratory in Public Health, England, 2012–2020
Source: Pathogens. 2023 Jan 31;12(2):223. doi: 10.3390/pathogens12020223 (PMC9966477; doi:10.3390/pathogens12020223)
Supplement: Supplementary file 1 [file pathogens-12-00223-s001.zip › Supplementary Table S1.pdf]

Supplementary Table S1 - Overview of published ECDC Risk assessments associated with *Salmonella* Outbreaks in England assessed by whole genome sequencing, 2012-February 2020

Date ROA/RRR Published	ECDC/ Urgent Inquiry No.	Date Range	No. Cases	Title	Summary	SNP Address
27-Aug-14	25-12-2014 ROA	June to August 2014	319	Multi-country outbreak of *Salmonella* Enteritidis infections associated with consumption of eggs from Germany	Sporadic or outbreak cases of *Salmonella* Enteritidis reported by Austria, France, Germany and the United Kingdom, in addition to one case reported in Luxembourg in a patient residing in France, appear to be linked by time of symptom onset and microbiological characteristics of isolates. isolates from contaminated eggs identified in France originating from the implicated German egg packaging<br>centre share similar molecular characteristics to the human cases. Isolates from a sample of a *Salmonella* contaminated strawberry cake, identified in Germany through an investigation unrelated to this outbreak, also<br>share similar molecular characteristics to the human cases.	1.2.3.18.175.175.%
06-Sep-16	29-08-2016-RRA	1 May 2016 to 30 August 2016<br><br>February 2012 to April 2016	16 Confirmed 132 Probable<br><br>101 Confirmed	Multi-country outbreak of *Salmonella* Enteritidis phage type 8, MLVA type 2-9-7-3-2 infections. First Update	A multi-country outbreak of *Salmonella* Enteritidis (phage type 8 with multiple-locus variable-number tandem repeat analysis profile 2-9-7-3-2) was analysed with whole genome sequencing methods. Despite the genetic characterisation of the pathogen, outbreak investigators have not been able to determine the source of the outbreak. Since May 2016, six EU/EEA countries have reported a total of 16 confirmed and 131 probable cases.	1.2.3.175.175.175.%<br>1.2.3.18.359.360.%
27-Oct-16	27-10-2016 ROA	1 May to 12 October 2016	112 Confirmed 148 Probable	Multi-country outbreak of *Salmonella* Enteritidis phage type 8, MLVA type 2-9-7-3-2 and 2-9-6-3-2 infections, 27 October 2016	From 1 May 2016 to 12 October 2016, seven EU/EEA countries have reported 112 confirmed cases belonging to two distinct WGS clusters, and 148 probable cases sharing the S. Enteritidis MLVA profiles 2-9-7-3-2 or 2-9-6-3-2. Outbreak cases, both confirmed and probable, have been reported by Belgium, Denmark, Luxembourg, the Netherlands, Norway, Sweden and the United Kingdom. Nine of the confirmed cases are associated with a travel history to Hungary or Poland, both of which countries are also considered to be affected by this outbreak. The available evidence from WGS, food and environmental investigations, as well as from tracing-back investigation of eggs, establishes a link between this multi-country foodborne outbreak and the packing centre B in Poland, pointing at eggs as the most likely vehicle of infection for at least part of the outbreak cases. Additional information from epidemiological, food and WGS investigations might bring further evidence on the possible vehicles and sources of infection associated with this outbreak.	1.2.3.175.175.175.%<br>1.2.3.18.359.360.%
01-Dec-16	05-12-2016 RRA	January 2012 to 24 November 2016	275	Multi-country outbreak of *Salmonella* Enteritidis PT8 infection, MLVA type 2-10-8-5-2, associated with handling of feeder mice	A persistent common-source, multi-country outbreak of *Salmonella* Enteritidis phage type (PT) 8 infection, characterised by MLVA type 2-10-8-5-2, has been ongoing in the United Kingdom (since at least 2011) and Denmark (since at least 2014). Cases are further defined through whole-genome-sequencing (WGS) analysis and are associated with exposure to pet reptiles, in particular corn snakes, and feeder mice. The British outbreak investigation team identified the feeder mice as being imported into the United Kingdom from a rodent farm in Lithuania. Additional EU/EEA countries where the implicated feeder mice were also distributed are likely to be affected by this outbreak.	1.5.159.280.280.280.%
06-Feb-17	06-02-2017-RRA	April 2014 - 26 January 2017	329 Confirmed 15 Probable	Re-emerging multi-country WGS-defined outbreak of *Salmonella* Enteritidis, MLVA type 2-12-7-3-2 and 2-14-7-3-2, 6 February 2017	The detection of a WGS-defined cluster of 17 cases of *S.* Enteritidis in the United Kingdom in the autumn of 2016 highlights the re-emergence of a strain that was first identified in 2014 and caused an outbreak that was investigated from May to October 2015 in Spain and the UK. This cluster is part of a multi-country outbreak involving at least four EU/EEA Member States, with 329 confirmed cases that has been ongoing since 2014 and it is being suggested that there might be a common source.	1.2.3.151.362.363.%
07-Mar-17	7-3-2017 ROA	May 2016 to 24 February 2017	218 confirmed 252 probable	Multi-country outbreak of *Salmonella* Enteritidis phage type 8, MLVA profile 2-9-7-3-2 and 2-9-6-3-2 infections, 7 March 2017	A multi-country outbreak of *Salmonella* Enteritidis phage type (PT) 8 with multiple locus variable-number tandem repeat analysis (MLVA) profiles 2-9-7-3-2 and 2-9-6-3-2, linked to eggs, is ongoing in the EU/EEA. Based on whole genome sequencing (WGS), isolates are part of two distinct but related genetic clusters. From 1 May 2016 to 24 February 2017, 14 EU/EEA countries have reported 218 confirmed cases belonging to two distinct WGS clusters, and 252 probable cases sharing the *S.* Enteritidis MLVA profiles 2-9-7-3-2 or 2-9-6-3-2. Outbreak cases, both confirmed and probable, have been reported by Belgium, Croatia, Denmark, Finland, France, Greece, Hungary, Italy, Luxembourg, the Netherlands, Norway, Slovenia, Sweden and the United Kingdom. Eleven confirmed cases are reported to have travelled to Poland during the incubation period. Poland is therefore likely to be affected by this outbreak as well. Croatia and Hungary reported a fatal case each.	1.2.3.175.175.175.%<br>1.2.3.18.359.360.%<br>1.2.3.18.455.469.%<br>1.2.3.18.455.2440.%

14-Jun-17	14-5-2017 ROA	May 2016 to 1 June 2018	47	Multi-country outbreak of new *Salmonella* enterica 11:z41:e,n,z15 infections associated with sesame seeds, 14 June 2017	An outbreak of a new serovar of *Salmonella* enterica subspecies enterica with antigenic formula 11:z41:e,n,z15, initially detected in Greece, affected 47 individuals in five European Union countries (the Czech Republic, Germany, Greece, Luxembourg and the United Kingdom) between March 2016 and May 2017. Epidemiological and microbiological evidence linked some of the outbreak cases to a sesame paste produced by a Greek manufacturer. The sesame seeds used for the production of the sesame paste were traced back to an African country. The same Greek manufacturer also processed a batch of sesame seeds imported from another African country that tested *Salmonella* positive after processing.	1.1.1.1.1.1.%
20-Jul-17	20-07-2017 RRA	May 2014 to 17 July 2017	314 confirmed<br>21 probable	Multi-country outbreak of *Salmonella* Enteritidis phage types 56 and 62, MLVA profile 2-11-3-3-2 and 2-12-3-3-2 infections, 20 July 2017	A multi-country outbreak of S. Enteritidis delineated through whole genome sequencing analysis is currently ongoing, with 314 confirmed cases in Austria, France, Ireland, Luxembourg and the United Kingdom. Confirmed cases belong to three closely related genetic clusters. Public Health England reports that investigations into these genetic clusters have shown that the outbreaks could be associated with the consumption of poultry products, i.e. meat or eggs.	1.2.3.151.151.151.%<br>1.2.3.151.151.718.%<br>1.2.3.151.151.783.%
12-Dec-17	12-12-2017-RRA	1 February 2017 to 28 November 2017	196 confirmed<br>72 probable	Multi-country outbreak of *Salmonella* Enteritidis infections linked to Polish eggs	A multi-country outbreak of *Salmonella* Enteritidis linked to eggs from Poland is ongoing in the EU/EEA. Testing through whole genome sequencing (WGS) shows the isolates are part of four distinct but related genetic clusters. ECDC and EFSA are liaising with relevant authorities in the affected EU/EEA countries and the European Commission to facilitate the coordination of investigation and response measures	1.2.3.175.175.175.%<br>1.2.3.18.359.360.%<br>1.2.3.18.455.469.%<br>1.2.3.18.455.2440.%
26-Jul-18	UI-478	1 January 2017 -<br>17 July 2018<br><br>Historical cases 2014-2016	122<br><br><br>25	Multi-country outbreak of *Salmonella* Agona infections possibly linked to ready-to-eat food	A multi-country outbreak of *Salmonella* enterica subspecies enterica serovar Agona (S. Agona) is under investigation in the European Union (EU), with cases retrospectively identified back to 2014. Overall, 147 outbreak cases have been reported by five EU countries, namely, Denmark, Finland, Germany, Ireland and the United Kingdom.	1.1.1.29.32.37.%
19-Nov-18	18-12-2018 ROA	February 2012 to 12 November 2018	1412	Multi-country outbreak of *Salmonella* Enteritidis infections linked to Polish eggs	Since the joint ECDC-EFSA rapid outbreak assessment 'Multi-country outbreak of *Salmonella* Enteritidis infections linked to Polish eggs' published on 12 December 2017, 15 EU/EEA countries (Belgium, Czech Republic, Denmark, France, Hungary, Iceland, Ireland, Italy, Luxembourg, Netherlands, Norway, Poland, Slovenia, Sweden and United Kingdom) have reported 336 confirmed, 94 probable and 3 new historical-confirmed cases associated with this ongoing multi-country outbreak of *Salmonella* Enteritidis in the EU/EEA. In the same period, seven historical probable cases were reclassified as excluded.	1.2.3.175.175.175.%<br>1.2.3.18.359.360.%<br>1.2.3.18.455.469.%<br>1.2.3.18.455.2440.%
06-Feb-20	UI-367	1 February 2017 -<br>14 January 2020	656	Multi-country outbreak of *Salmonella* Enteritidis infections linked to eggs - Third update	A multi-country outbreak of *Salmonella* Enteritidis, delineated by whole genome sequencing (WGS), linked to eggs, has been ongoing in the EU/EEA for several years. From 1 February 2017 to 14 January 2020, 15 EU/EEA countries reported 656 confirmed cases and 202 probable cases. Before February 2017, 385 historical-confirmed cases and 413 historical-probable cases were identified, resulting in 18 affected countries. Due to differences in capacity for case confirmation, more countries are likely to be affected.	1.2.3.175.175.175.%<br>1.2.3.18.359.360.%<br>1.2.3.18.455.469.%<br>1.2.3.18.455.2440.%
21-Oct-20	UI-636	1 August 2019 - 20 October 2020	123	Multi-country outbreak of *Salmonella* Typhimurium and S. Anatum infections linked to Brazil nuts	123 cases of *S.* Typhimurium ST19 and one case of *S.* Anatum have been reported, of which 105 were in the UK (including the *S.* Anatum case), 14 in France, three in Luxembourg, one each in the Netherlands and Canada. A case-control study in the UK and patient interviews in the UK, France and Luxembourg indicated Brazil nuts and nut bars as likely vehicles of infections.	4.123.150.180.205.214.%
25-Feb-21	UI- 644	May 2018 - December 2020	193	Multi-country outbreak of *Salmonella* Enteritidis sequence type (ST)11 infections linked to poultry products in the EU/EEA and the United Kingdom	*Salmonella* Enteritidis sequence type (ST)11 were reported in Denmark (2), Finland (4), France (33), Germany (6), Ireland (12), the Netherlands (3), Poland (5), Sweden (6), and the United Kingdom (UK) (122). One in five cases was hospitalised. The most recent case was reported by the UK in December 2020. Epidemiological studies in the UK have identified an increased risk of *S.* Enteritidis infection associated with the consumption of frozen breaded chicken products. Fifty percent of the cases were children ≤ 18 years. Multiple other serovars have also been found in food products including S. Newport, S. Infantis, S. Java and S. Livingstone)	1.1.2.2533.3617.4833.%<br>(1.1.2.12.%)<br>(1.2.3.18.180.180.%)<br>(1.1.2.12.12.590.%)<br>(1.1.2.28.195.6669.%)

Summary Overview of Rapid Outbreak Assessments (ROA) or Rapid Risk Assessments (RRR) between ECDC, EFSA, UKHSA and other international reference centres. No. of cases correct at time of publication of the risk assessment.
